# Rapid Detection of Transition Metals in Welding Fumes Using Paper-Based Analytical Devices

**DOI:** 10.1093/annhyg/met078

**Published:** 2014-02-10

**Authors:** David M. Cate, Pavisara Nanthasurasak, Pornpak Riwkulkajorn, Christian L’Orange, Charles S. Henry, John Volckens

**Affiliations:** ^1.^Department of Biomedical Engineering, Colorado State University, Fort Collins, CO 80523, USA; ^2.^Department of Chemistry, Faculty of Science, Chulalongkorn University, Pathumwan, Bangkok 10330, Thailand; ^3.^Department of Environmental and Radiological Health Sciences, Colorado State University, Fort Collins, CO 80524, USA; ^4.^Department of Chemistry, Colorado State University, Fort Collins, CO 80523, USA

**Keywords:** µPADs, chromium, colorimetric detection, exposure, iron, low cost, nickel

## Abstract

Metals in particulate matter (PM) are considered a driving factor for many pathologies. Despite the hazards associated with particulate metals, personal exposures for at-risk workers are rarely assessed due to the cost and effort associated with monitoring. As a result, routine exposure assessments are performed for only a small fraction of the exposed workforce. The objective of this research was to evaluate a relatively new technology, microfluidic paper-based analytical devices (µPADs), for measuring the metals content in welding fumes. Fumes from three common welding techniques (shielded metal arc, metal inert gas, and tungsten inert gas welding) were sampled in two welding shops. Concentrations of acid-extractable Fe, Cu, Ni, and Cr were measured and independently verified using inductively coupled plasma-optical emission spectroscopy (ICP-OES). Results from the µPAD sensors agreed well with ICP-OES analysis; the two methods gave statistically similar results in >80% of the samples analyzed. Analytical costs for the µPAD technique were ~50 times lower than market-rate costs with ICP-OES. Further, the µPAD method was capable of providing same-day results (as opposed several weeks for ICP laboratory analysis). Results of this work suggest that µPAD sensors are a viable, yet inexpensive alternative to traditional analytic methods for transition metals in welding fume PM. These sensors have potential to enable substantially higher levels of hazard surveillance for a given resource cost, especially in resource-limited environments.

## INTRODUCTION

Human exposure to metal-containing particulate matter (PM) in industries such as mining, construction, and manufacturing significantly impacts worker health. Occupational respiratory diseases cost ~$10B each year in the USA and result in ~425000 premature deaths annually worldwide (Nelson *et al*., 2005). Known pathologies include pneumoconiosis (Della Torre *et al*., 1990; Frank *et al*., 2012), respiratory and cardiovascular impairment (Gavett and Koren, 2001; Ibfelt *et al*., 2010; Phillips *et al*., 2010; Szram *et al*., 2013), ‘metal fume fever’ (Cain and Fletcher, 2010; Kunimasa *et al*., 2011; Mehta *et al*., 2012), and lung cancer (Jomova and Valko, 2011; Zeidler-Erdely *et al*., 2011; ‘t Mannetje *et al*., 2012). Of particular concern is exposure to welding fumes, known to contain hazardous levels of particulate metals such as hexavalent chromium, nickel, copper, nitrous oxide, manganese, and lead (Quansah and Jaakkola, 2009). Despite the risks posed by these inhalation hazards, welders’ exposure to particulate metals is infrequently assessed due to the high cost and effort associated with personal exposure measurement [US Department of Labor (USDOL), 2008]. Regulatory compliance monitoring for welding fumes calls for an 8-h filter sample (collected within the worker’s breathing zone) followed by chemical analysis via flame atomic absorption or inductively coupled plasma emission spectrometry (USDOL, 2007). Both of these techniques require large and expensive instrumentation and highly trained staff, resulting in analysis costs of >$100 per sample (depending on the number of analytes measured); these costs include sample preparation, sample analysis, and personnel time. In the developed world, such costs tend to preclude routine exposure assessments; in the developing world, these costs render the exposure assessment practically impossible. Furthermore, because collected samples must be shipped to a central laboratory for analysis, the time from sample collection to reporting (i.e. hazard communication) is typically on the order of several weeks. Consequently, there is a need for simple, sensitive, and cost-friendly alternatives for monitoring workers’ exposure to PM metals that would enable broader screening of occupational exposures (Leman *et al*., 2010; Fierz *et al*., 2011). This need is particularly evident since such exposures tend to be spatiotemporally variable and log-normally distributed (Flynn and Susi, 2010).

There is growing demand for new exposure measurement approaches that are both affordable and available for use at the point of need. An emerging technology that may address this demand is microfluidic paper-based analytical devices (µPADs) (Khan *et al*., 2010; Li *et al*., 2012; Su *et al*., 2012)—a new technology platform for extremely low-cost sensing applications. The µPAD concept is similar to the ‘lab-on-a-chip’ notion, but where the ‘chip’ is replaced with simple cellulosic paper. In a typical µPAD, hydrophobic barriers, printed onto the paper, define fluidic circuits that control liquid (sample) transport. These fluidic circuits are chemically modifiable and, therefore, amenable to a variety of physical, chemical, and biological measurement applications (Belgacem *et al*., 2011). Relative to traditional chemical assays, µPADs require low reagent volumes (typically microliters), are simple to operate, portable, and inexpensive (Martinez *et al*., 2010; Ballerini *et al*., 2012; López Marzo *et al*., 2013; Zwanenburg *et al*., 2013). Even at low production numbers, these devices often cost <$0.05 to produce. As a consequence of using small sample volumes, mass-based detection sensitivities in paper devices are often comparable to or better than analogous detection moieties in traditional assays (Liu *et al*., 2011; Wang *et al*., 2012).

One of the most common techniques for quantifying analytes on paper is colorimetry. Colorimetric sensors are attractive for analytical measurements because they offer a high-contrast signal that is easy to quantify with an external optical reader such as a scanner, camera, or smartphone (Yang *et al*., 2005; Martinez *et al*., 2008; Zhao *et al*., 2008; Ellerbee *et al*., 2009; Jokerst *et al*., 2012; Sameenoi *et al*., 2013). Several reports have focused on the detection of metals in water using nanoparticle aggregation (Hossain and Brennan, 2011; Yang and Wang, 2011; Lafleur *et al*., 2012; Ratnarathorn *et al*., 2012; Ferhan *et al*., 2013) and enzymatic action (Ratnarathorn *et al*., 2012). Our group was one of the first to extend colorimetry to paper devices for the measurement of Fe, Cu, and Ni in combustion ash samples (Mentele *et al*., 2012). Functionalized Ag nanoparticles have been used to measure Cu on paper substrates with a reported linear detection range of 8–62 µM (Ratnarathorn *et al*., 2012). Lateral flow chromatography systems have also been developed for measuring Cu, Cr, and Ni with 0.02, 0.15, and 0.23 µg ml^−1^ detection sensitivities, respectively (Hossain and Brennan, 2011).

The objective here is to extend the application of colorimetric µPADs to welding fumes and to the detection of total Cr. To demonstrate the utility of our method, we developed a paper sensor capable of measuring concentrations of acid-soluble Fe, Ni, Cu, and Cr with punches taken from air sampling filters. A series of filter samples was taken at various welding facilities and analyzed concurrently using the µPAD method and a standard technique: inductively coupled plasma-optical emission spectroscopy (ICP-OES). Samples were collected from three separate welding processes [tungsten inert gas (TIG), metal inert gas (MIG), and shielded metal arc welding (SMAW)] with several common SAE stainless steel (SS) grades (304, 308, 309, and 17-4 PH). Analytical costs to quantify concentrations of 28 analytes were on the order of $20 for the µPAD, compared to ~$1000 using laboratory ICP-OES. Of the 28 paired measurements, 20 were not statistically different from each other at the 95% confidence interval; the remaining eight samples gave a mean difference (µPAD versus ICP-OES) of <20%.

## METHODS

### Chemicals and materials

All chemicals were analytical grade and used as received without further purification. Iron(III) chloride hexahydrate, nickel(II) sulfate hexahydrate, aluminum(III) sulfate hydrate, copper(II) sulfate pentahydrate, phthalic anhydride, dimethylglyoxime (DMG), sodium acetate trihydrate, sodium fluoride, cerium(IV) ammonium nitrate, 1,5-diphenylcarbazide (1,5-DPC), and polydiallyldimethylammonium chloride (PDDA, medium molecular weight) were purchased from Sigma-Aldrich (St Louis, MO, USA). Tris-hydrochloride and ammonium hydroxide were purchased from Mallinckrodt Baker, Inc. (Phillipsburg, NJ, USA). Glacial acetic acid was purchased from Fisher Scientific (Pittsburgh, PA, USA). Nitric acid (18.4M) was purchased from EMD Millipore (Billerica, MA, USA). Milli-Q water from a Millipore deionized water generator (*R* ≥ 18.2 MΩ cm^−1^) was used for all experiments. Mixed cellulose ester (MCE) filters were purchased from Fisher Scientific Company (Pittsburgh, PA, USA). Whatman No. 1 qualitative-grade filter paper was purchased from General Electric Company (Schenectady, NY, USA).

### Welding fume sampling

Samples were collected from SMAW, MIG, and TIG welding processes. Each welding technique used a different stainless steel alloy of varying composition of Cr, Fe, Cu, and Ni (Table 1 provides composition information per the manufacturer). Specifically, 304 SS was used for TIG welding; alloys of 304, 309-EL, and 17-4 PH were used for SMAW; and 304, 308, and 17-4 PH SS alloys were used for MIG welding. Area samples were taken on multiple days in the vicinity of each welding operation. Aerosol was sampled onto 37-mm MCE filters (0.8 µm pore size) using a size-selective sampling cassette (PM_10_ PEM; SKC, Fullerton, CA, USA) designed to collect particles <10 µm in aerodynamic diameter. The sample air flow rate was set to 4 l min^−1^ and sampling duration lasted ~8h. In total, 15 filters were collected, extracted, and analyzed. Method validation was performed independently by inductively coupled plasma-optical emission spectroscopy (ICP-OES) on seven 10-mm punches taken from 37-mm diameter filters (Technology Laboratories, Fort Collins, CO, USA). In order to compare both µPAD and ICP methods, filter punches were analyzed from the same filter. One of our assumptions was that PM was homogeneously distributed across the filter because control samples from the same filter (tested by ICP-OES) differed by <0.01 µg for each metal (data not shown). Sample preparation and ICP-OES analysis followed EPA (Environmental Protection Agency) Methods 3050B and 6010B, respectively. Metal content on the field blank filters was below the detection limit of the ICP instrument.

**Table 1.** Percent composition of Ni, Cr, Cu, and Fe in the stainless steel alloys (SAE grade) used for collecting of welding fumes. Table information was provided by the manufacturer

Alloy	% Nickel	% Chromium	% Copper	% Iron
304	8–10.5	18–20	0–1	>50
308	10–12	19–21	Trace	>50
309-EL	12–15	22–24	Trace	>50
17-4 PH	3–5	15–17.5	3–5	>50

### µPAD fabrication and colorimetric assay

Paper devices were designed in CorelDraw and Adobe Illustrator and fabricated as described in Fig. 1a. Briefly, wax barriers were printed onto filter paper using a commercial wax printer (Xerox Colorqube 8870); these barriers were then melted into the paper (creating a 3D hydrophobic channel) by placing the paper onto a 150°C hotplate for 60 s (Ge *et al*., 2012a,b). After cooling, packing tape was applied to one side of the filter paper to prevent reagents from leaking through the device. A picture of the device is provided (see Supplementary Figure 1, available at *Annals of Occupational Hygiene* online). Previously described reagent deposition protocols for detection of Fe, Ni, and Cu were followed (Mentele *et al*., 2012). For determination of total acid-extractable Cr, 0.5-µl ceric(IV) ammonium nitrate (0.35mM) was first added to the pretreatment zone twice, followed by 0.5 µl of PDDA (5% w/v). PDDA was necessary to stabilize the Cr–1,5-DPC reaction product and decrease the mobility of the product complex in the detection zone (Xiao *et al*., 2012). A mixture of 15mg ml^−1^ 1,5-DPC and 40mg ml^−1^ phthalic anhydride was prepared in acetone and deposited once on the detection zone (0.5 µl). The pretreatment and detection zones were dried between additions of reagent. In the presence of Cr(VI), the colorimetric reagent 1,5-DPC becomes oxidized to diphenylcarbazone, reacting with trivalent Cr to produce an intensely purple-hued complex (Fig. 1b) (Kong, 2009; Kong and Ni, 2009).

**1:**
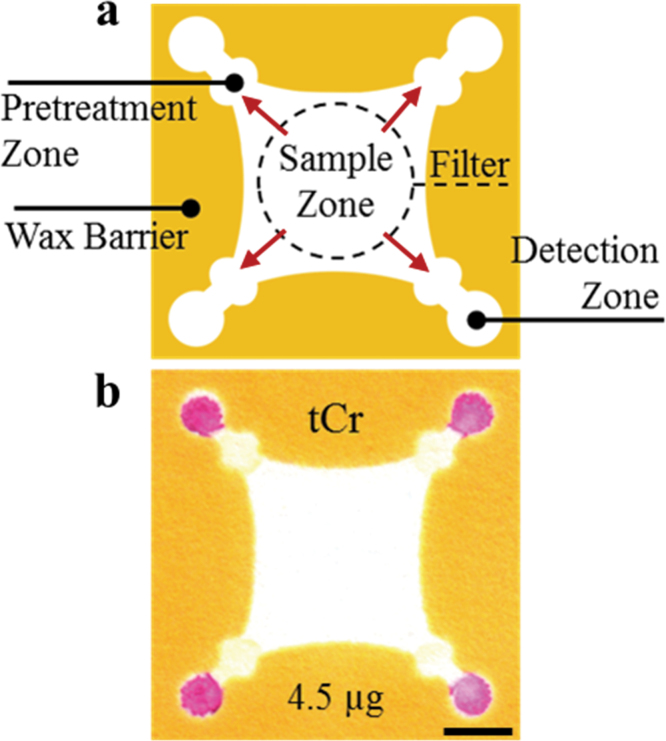
Schematic showing specific zones within a µPAD. (a) A filter punch is placed on the sample zone where metals are eluted off the filter, onto the sample zone, and then outwards through the pretreatment and detection zones (red arrows). Reagents deposited in the pretreatment zone control solution pH and complex interferences that may be present in the sample. Colorimetric reagents in the detection zone complex with each metal analyte. The filter and device can be thrown away after use. (b) Image of actual device used for the detection of total acid-extractable Cr. The scale bar represents 5mm.

### Filter extraction and µPAD analysis

Following sample collection, 10-mm punches were taken from each filter and subjected to microwave-assisted acid digestion (Fig. 2). Wetting and extraction efficiency was enhanced by pipetting 20 µl of surfactant [sodium dodecyl sulfate (SDS), 5 mM] onto each punch followed by air drying prior to sample digestion. To digest the metals in the welding fume, 5 µl of concentrated HNO_3_/SDS (5mM) was added to the punch along with 30 µl deionized water. Each punch was placed in a microwave (1100W) for 15 s. A second water/SDS (5mM) mixture (30 µl) was again added to the punch (to keep the filter wet), followed by another 15 s in the microwave; this wetting/microwave step was repeated twice. After digestion, the filter punch was neutralized by adding 10 µl of sodium bicarbonate (0.5M, pH 9.5), dried, and placed on the sample zone of the µPAD. For each test, a poly(dimethylsiloxane) (PDMS) lid, designed to reduce eluent evaporation and to distribute pressure evenly across the paper surface, was placed on top of filter punch/µPAD. The lid also contained openings above the sample (3mm diameter) and detection (5mm diameter) zones for solvent/buffer addition. Acetate buffer (40 µl, 0.1M, pH 4.5) was next added to the sample zone and a 300g weight was placed on the PDMS lid to help stabilize flow across the device. Metal detection was accomplished in ~20min after the eluent had completely dried. Devices were then analyzed using a common flatbed scanner.

**2:**
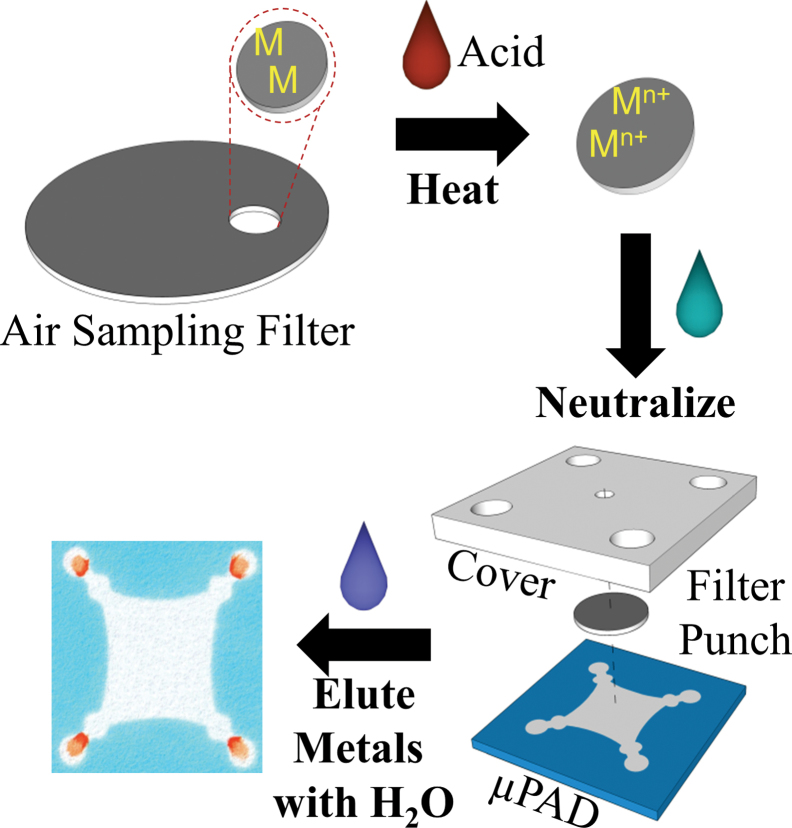
Representative schematic demonstrating steps to measure particulate metals. A 10-mm filter punch is subjected to microwave acid digestion, neutralized, and water is used to elute metals from the filter punch outward to detection zones at the periphery. µPAD dimensions are ~2.7×2.7cm. A PDMS cover is used to control addition of eluent and to displace liquid evenly across the device.

### Image processing

For quantitation, devices were scanned using a desktop flatbed scanner (XEROX DocuMate 3220), providing a high resolution, well-focused image. This detection method was chosen because office scanners are available worldwide and because scanned images are typically unaffected by external lighting conditions. A color thresholding window was applied to each image using ImageJ software (Rasband, 2009) to remove background interferences from the paper. Wax backgrounds were colored in hues that are complementary to the hue of the analyte complex being measured. A complementary-colored background was easiest to remove using the thresholding window. Image intensity units selected for the thresholding window for each metal were: Fe = 18–230 PIU, Ni = 10–210 PIU, Cu = 35–225 PIU, and Cr = 0–180 PIU. Pure white and black backgrounds were considered 255 and 0 pixel intensity, respectively. After thresholding, images were inverted and the color intensity at each detection zone was measured as the arithmetic mean of pixel intensity. Measurements from all four detection zones were then averaged to yield a single result for each metal of interest.

## Results

### Metal determination using µPADs

Calibration curves and analytic figures of merit were generated for each metal of interest. Iron was measured by the intensity of the reddish ferroin complex [Fe(phen)_3_]^2+^ after complexation with 1,10-phenanthroline (Brandt *et al*., 1954). The detection limit for Fe was 1.1 µg with a linear range between 1.1 and 10 µg (Fig. 3a) with a relative standard deviation (SD) of 7.7% (number of samples *n* ≥ 4). Above 10 µg Fe, the color signal begins to saturate and a detection threshold is reached around 15 µg. Further increases in linear range could be achieved using different sized detection zones but this step was not required here. The range of measurement for µPAD-based quantification of Fe is 7.8–107 µg m^−3^ as a time-weighted average (TWA) air concentration (based on a 4 l min^−1^ sample collected over 8h). We also tested the interdevice variability of our method with Fe and Ni as the analyte (see Supplementary Figure 2, available at *Annals of Occupational Hygiene* online). Over the course of 3 weeks, calibration curves were generated across the working range of the assay. Reagents were made fresh each time. For Fe and Ni, the average difference in the slope for all linear fits was 4.8±4.4% and 9.7±5.2%, respectively. The average difference in measured intensity [in pixel intensity units (PIU)] per mass of metal for Fe and Ni was 5.6±5.7 and 1.5±0.59 PIU, respectively.

**3:**
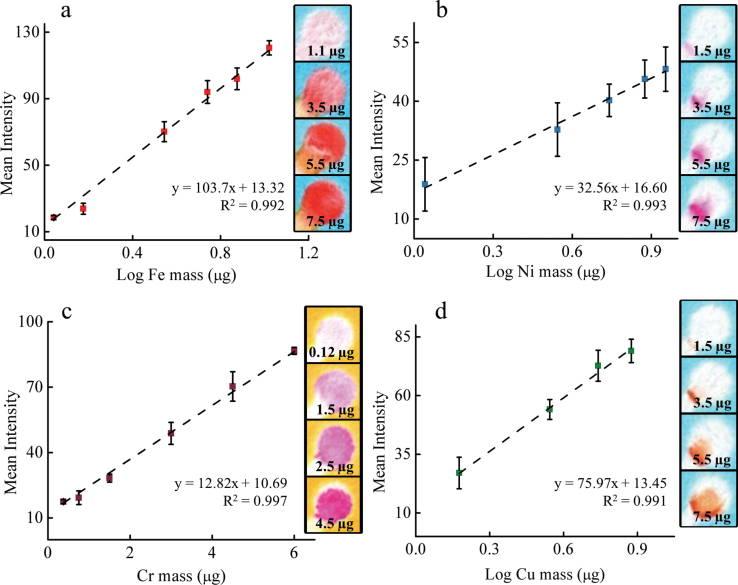
Typical response curves obtained for the measurement of acid-extractable (a) iron, (b) nickel, (c) chromium, and (d) copper. The linear response generated for each metal was 1.1–10 µg Fe, 1–10 µg Ni, 0.15–6 µg Cr, and 1.5–8 µg Cu. µPAD error bars are based on measurements between samples (N ≥ 4). Colorimetric intensity was determined using a desktop scanner. Raw samples images for each metal are included to the right of each calibration graph.

Nickel was measured by recording the intensity of the magenta-colored complex formed by reaction with DMG (Fig. 3b) (Booth and Strickland, 1953). Acetic acid and NaF were added to the pretreatment zone as masking agents for Fe, Cu, and Co; as a result, these metals are sequestered upstream of the Ni detection zone. Analyte intensity was log linear with respect to Ni mass with a dynamic range of 1.1–9.0 µg (7.8–64.2 µg m^−3^ TWA) and a relative SD of 17.9% (*n* = 4). There was no discernible color produced when a single filter punch was analyzed, so two punches were stacked, placed in the sample zone of the device, and analyzed (Table 2). While this method decreases analytical precision somewhat, the interdevice relative SD remains <20%. The largest detectable mass prior to color intensity saturation was 20 µg, however this upper limit was outside the dynamic linear range of the test (data not shown).

**Table 2.** Chemical validation by ICP-OES compared to µPAD-measured metal content for each 10-mm filter punch. Particulate metal samples from three different welding processes (TIG, SMAW, and MIG) were collected and evaluated. Cu was not detected in any sample with either the µPAD or the ICP instrument (0.05 µg limit of detection). The content of all four transition metals of a filter blank was below ICP detection limits

Metal	Welding type	ICP-OES ± SD (µg)	µPAD ± SD (µg)	Recovery %	Relative standard deviation_µPAD_ %
Fe	SMAW	9.52±1.43	9.45±1.00	99.3	10.6
	MIG	2.77±0.42	2.25±0.07^a^	81.2	3.15
	TIG	1.55±0.23	1.21±0.11^a^	78.1	9.42
Ni	MIG^b^	1.53±0.23	1.50±0.39	98.0	25.7
Cr	SMAW 1^b^	2.01±0.30	1.81±0.44	90.0	24.4
	SMAW 2^c^	0.86±0.13	0.81±0.35	94.0	43.8
	MIG	0.36±0.05	0.41±0.06	115.0	13.8
Cu		<0.05	Too low	—	—

^a^Two of the tested samples were statistically different than metal content measured by ICP-OES.

^b^Three filter punches used for analysis.

^c^Two filter punches used for analysis.

Total, acid-extractable Cr was measured using 1,5-DPC as the detection reagent (Fig. 3c) (Farag *et al*., 1981). The measured intensity was linear with respect to Cr mass with a range between 0.37 and 6 µg and a relative SD of 8.2% among repeated measurements. Three filter punches were stacked and analyzed simultaneously for one of the SMAW samples (Table 2), but only one punch was used for each of the other two welding fume samples for which Cr was measured. This method provided quantitative measurements of Cr air concentrations (based on an 8-h TWA at a 4 l min^−1^ sample flow) in the range of 2.6–42.8 µg m^−3^. The detection limit was almost an order of magnitude lower for Cr than for Fe, due in part to the larger molar absorptivity of the Cr–1,5-DPC product (4.6×10^4^ l mol^−1^ cm^−1^) compared to Fe–1,10-phenanthroline (1.1×10^4^ l mol^−1^ cm^−1^) (Xu *et al*., 1996; Filik *et al*., 2003).

For the detection of copper, bathocuproine was used to produce an orange-brown complex with Cu^2+^ (Fig. 3d) (Wilkins and Frederick Smith, 1953; Penner and Inman, 1963; Mentele *et al*., 2012). We were able to detect Cu reproducibly at masses as low as 1.5 µg. The dynamic range for analyte measurement as a TWA is 10.7–121.2 µg m^−3^. The personal exposure limit (PEL) for Cu exposure is 100 µg m^−3^, which is within the dynamic range of the test. However, Cu was not detected on any field samples by either the ICP-OES or paper-based methods.

The PAD sensors are stable when stored (in the dark and at 25°C) for 7–30 days, depending on which reagents are added to the PAD. The PADs for detection of Cr can be stored and used for up to 30 days, whereas the PADs for Cu can be stored for up to 7 days without significant loss of functionality (Rattanarat *et al*., 2013).

### Method validation

Filter samples were collected from three SS welding processes (TIG, MIG, and SMAW) using three of the most commonly used SS alloys (304, 309, and 17-4 PH) in the welding industry. Levels of Fe, Ni, Cu, and total (acid extractable) Cr were then quantified using both µPAD and ICP-OES methods. In total, 28 analytes were measured from seven filter punches. Results of these tests are presented in Table 2 and shown in a 1:1 plot in Fig. 4. Metals measured on five of seven filter samples were not statistically different at the 95% confidence level. Additionally, the µPAD method was sensitive, with >90% accuracy for 20 analytes. Two of the Fe samples (MIG and TIG) were statistically different (µPAD versus ICP-OES), however the Fe levels reported by the µPAD were within 20% of the ICP-OES method. Detectable levels of Cu were not seen using either method, indicating that Cu levels were below the detection limit of the ICP instrument (0.4 µg l^−1^). This result was not surprising because Cu is only present in significant quantity in few SS alloys and as a result, personal exposure to Cu is not considered a primary danger to welders.

**4:**
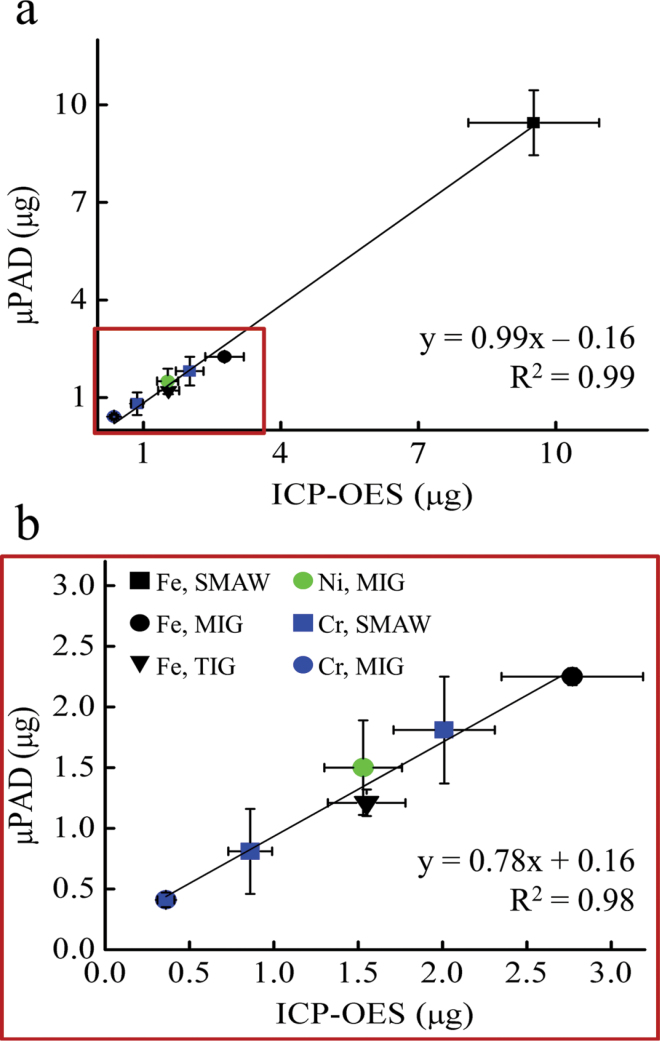
(a) Paper-based measurement of Fe, Ni, and Cr compared with independent validation by ICP-OES. A linear regression fit favorably compares the accuracy of the µPAD method with ICP-OES (slope = ~0.99). Cu was present below the detection limits of both measurement methods and was thus not presented. (b) An expanded view of the graph above shows that on small a smaller mass scale, method correlation is slightly worse (*R* ^2^ = ~ 0.98), however this value is still within acceptable limits.

## DISCUSSION

This is the first reported method, to our knowledge, for speciating hazardous workplace aerosols quickly and at low cost. Similar to gas detector tubes, our method requires little lab turnaround time, is simple to operate, and was developed specifically to make exposure assessment quicker, easier, and cost effective. The dynamic range of our screening method (two orders of magnitude) does not yet match traditional instrumentation such as ICP, however there remains great potential for µPAD techniques to offer rapid, on-site analysis (similar to gas detection tubes) enabling more frequent and thorough monitoring of occupational environments.

Currently, the cost and time associated with occupational exposure assessment represent a significant impediment towards adequate hazard surveillance. According to the Department of Labor, in 2012, there were ~329710 welders, cutters, solderers, and brazers in the USA (Bureau of Labor Statistics, 2012; USDOL, 2008). The analytical costs (not including personnel time and equipment) to assess each individual’s exposure just once and to a single metal species would exceed $33000000 per year. In contrast, the µPAD method described here has the potential to reduce analytical costs by a factor of 50. This method is also amenable for rapid, on-site detection immediately following sample collection. Results presented here are encouraging, from the standpoints of detection sensitivity, method repeatability, and accuracy.

The linear detection range for Fe is sufficient for occupational exposure assessment, given that the PEL stipulated by the Occupational Safety and Health Administration (OSHA) is 10000 µg m^−3^ for Fe. Several assays can be performed from a single 37-mm MCE filter (each test consumes a single 10-mm punch) should it be necessary to re-evaluate filter samples (or send samples to a lab for independent validation). We were not able to assess method accuracy for Cu, since all samples were below the detection limit. For Fe, Ni, and Cu, the relationship between colorimetric intensity and analyte mass is presented as log linear due to the characteristics of color saturation on paper. It is important to note that the dynamic ranges reported in Fig. 3 do not represent the entire dynamic range of the method, but only the range necessary for the processed samples.

The dynamic range of the µPAD method (for all four analytes) reported here is constrained by molar absorptivity of the colorimetric reagent (lower end) and signal saturation (upper end), highlighting a limitation of colorimetric sensing on paper. When the paper surface becomes saturated with analyte (as is the case in the detection zones), the resulting intensity reaches a threshold limit. Although the detection limits reported here are adequate for monitoring personal exposure at levels below the OSHA-regulated limits, a larger dynamic detection range may be desired, especially for short-term sampling. The upper end of the dynamic range may be extended by increasing the size of the detection zone; larger paper surface area equates to slower surface saturation (more area for color development). In addition, multiple punches may be analyzed simultaneously (i.e. stacked onto the µPAD) to extend the detection limit to lower masses (data not shown). Yet another option for improving device sensitivity is to design a µPAD with multiple detection zones of varying size.

Although we report total Cr mass here, the 1,5-DPC reagent is specific to hexavalent chromium (Cr(VI)) and thus, future application of this technology could assess exposures to the more toxic Cr(VI). Unfortunately, the ICP validation method we chose could not speciate between different Cr oxidation states. To measure Cr(VI) via µPAD, the same protocol could be followed, but tetravalent cerium (Ce(IV)) could be excluded from the pretreatment zone. Tetravalent cerium oxidizes soluble Cr(III) to Cr(VI) for complexation with 1,5-DPC, and thus, in the absence of Ce(IV), only Cr(VI) from the original sample is measured (Farag *et al*., 1981). As a result, this method shows promise for measurement of total soluble Cr or soluble Cr(VI). Future work will investigate Cr speciation using µPADs.

Several interfering metals present in welding fumes have the ability to complex with the chromophores in the detection zones. In previous work, we discussed our approach to ‘masking’ these interferences on paper using pretreatment zones (Mentele *et al*., 2012). For Cr(VI), we investigated potential interferences from Mg, Mn, Zn, Al, Ba, V, Co, Cu, Fe, and Ni and found that there was no significant effect for determination of Cr(VI) when other metals were present in metal:Cr(VI) ratios <4:1 (data not shown). This concentration ratio is reasonable for welding fume because Fe is usually the largest constituent in most welding fumes and is almost never present at four times the concentration of Cr, which typically represents 15–22% of welded metals. Examination of the 1:1 plot in Fig. 4 indicates that interferences from other metals presented only a minor influence for analyte detection on paper, if at all. Additionally, no significant differences were detected in device sensitivity when Ni and Fe were measured using 40 different devices over a 3-week period (Supplementary Figure 2, available at *Annals of Occupational Hygiene* online).

## CONCLUSIONS

The µPAD presented here offers a much simpler and less expensive alternative for measuring human exposure to toxic metals than current methods. Colorimetric detection provides a convenient, portable, and rapid way to quantify exposure at the point of need, whereas current methods require more expensive and lengthy, offsite analyses. This µPAD sensor enables sensitive determination of Fe, Cu, Ni, and Cr for ~50 times less cost than ICP-based methods. Consequently, µPADs show great potential as an enabling technology for a new wave of low-cost, high-throughput sensing platforms. Future work will focus on method modifications to improve sensitivity, quantitative range, and functionality, in addition to extending this method to other species. Ultimately, paper-based sensors may enable more comprehensive hazard recognition and surveillance worldwide. This technology appears well suited for resource-limited environments, where improvements in workplace safety can be challenging. However, several obstacles must be overcome before exposure assessment costs are low enough for more widespread sampling and analysis; for example, the costs associated with personal sampling pumps and size-selective inlets are still relatively high.

## SUPPLEMENTARY DATA

Supplementary data can be found at http://annhyg.oxfordjournals.org/.

## FUNDING

National Institute for Occupational Safety and Health (OH010050); National Institute of Environmental Health Sciences (ES019264).

## Supplementary Material

Supplementary Data

## References

[CIT0001] Ballerini D Li X Shen W (2012) Patterned paper and alternative materials as substrates for low-cost microfluidic diagnostics. Microfluid Nanofluid; 13: 769–87

[CIT0002] Belgacem MN Salon-Brochier MC Krouit M (2011) Recent advances in surface chemical modification of cellulose fibres. J Adhes Sci Technol; 25: 661–84

[CIT0003] Booth E Strickland J (1953) The compounds formed between nickel (II) and dimethylglyoxime by alkaline oxidation. J Am Chem Soc; 75: 3017–19

[CIT0004] Brandt WW Dwyer FP Gyarfas ED (1954) Chelate complexes of 1,10-phenanthroline and related compounds. Chem Rev; 54: 959–1017

[CIT0005] Bureau of Labor Statistics Occupational employment and wages Available at http://www.bls.gov/oes/current/oes514121.htm

[CIT0006] Cain JR Fletcher RM (2010) Diagnosing metal fume fever–an integrated approach. Occup Med (Lond); 60: 398–400 20407044 10.1093/occmed/kqq036

[CIT0007] Della Torre F Cassani M Segale M (1990) Trace metal lung diseases: a new fatal case of hard metal pneumoconiosis. Respiration; 57: 248–53 2095607 10.1159/000195850

[CIT0008] Ellerbee AK Phillips ST Siegel AC (2009) Quantifying colorimetric assays in paper-based microfluidic devices by measuring the transmission of light through paper. Anal Chem; 81: 8447–52 19722495 10.1021/ac901307q

[CIT0009] Farag A El-Wakil A El-Shahawi M (1981) Qualitative and semi-quantitative determination of chromium (VI) in aqueous solution using 1, 5-diphenylcarbazide-loaded foam. Analyst; 106: 809–12

[CIT0010] Ferhan AR Guo L Zhou X (2013) Solid-phase colorimetric sensor based on gold nanoparticle-loaded polymer brushes: lead detection as a case study. Anal Chem; 85: 4094–9 23509859 10.1021/ac4001817

[CIT0011] Fierz M Houle C Steigmeier P (2011) Design, calibration, and field performance of a miniature diffusion size classifier. Aerosol Sci Tech; 45: 1–10

[CIT0012] Filik H Doğutan M Apak R (2003) Speciation analysis of chromium by separation on a 5-palmitoyl oxine-functionalized XAD-2 resin and spectrophotometric determination with diphenylcarbazide. Anal Bioanal Chem; 376: 928–33 12819851 10.1007/s00216-003-2006-y

[CIT0013] Flynn MR Susi P (2010) Manganese, iron, and total particulate exposures to welders. J Occup Environ Hyg; 7: 115–26 20013450 10.1080/15459620903454600

[CIT0014] Frank H Monika R-H Tobias W (2012) Relation between biomarkers in exhaled breath condensate and internal exposure to metals from gas metal arc welding. J Breath Res; 6: 027105 22622358 10.1088/1752-7155/6/2/027105

[CIT0015] Gavett SH Koren HS (2001) The role of particulate matter in exacerbation of atopic asthma. Int Arch Allergy Immunol; 124: 109–12 11306943 10.1159/000053685

[CIT0016] Ge L Yan J Song X (2012a) Three-dimensional paper-based electrochemiluminescence immunodevice for multiplexed measurement of biomarkers and point-of-care testing. Biomaterials; 33: 1024–31 22074665 10.1016/j.biomaterials.2011.10.065

[CIT0017] Ge S Ge L Yan M (2012b) A disposable paper-based electrochemical sensor with an addressable electrode array for cancer screening. Chem Commun (Camb); 48: 9397–9 22889917 10.1039/c2cc34887j

[CIT0018] Hossain SM Brennan JD (2011) β-Galactosidase-based colorimetric paper sensor for determination of heavy metals. Anal Chem; 83: 8772–8 22029903 10.1021/ac202290d

[CIT0019] Ibfelt E Bonde JP Hansen J (2010) Exposure to metal welding fume particles and risk for cardiovascular disease in Denmark: a prospective cohort study. Occup Environ Med; 67: 772–7 20581417 10.1136/oem.2009.051086

[CIT0020] Jokerst JC Adkins JA Bisha B (2012) Development of a paper-based analytical device for colorimetric detection of select foodborne pathogens. Anal Chem; 84: 2900–7 22320200 10.1021/ac203466y

[CIT0021] Jomova K Valko M (2011) Advances in metal-induced oxidative stress and human disease. Toxicology; 283: 65–87 21414382 10.1016/j.tox.2011.03.001

[CIT0022] Khan MS Thouas G Shen W (2010) Paper diagnostic for instantaneous blood typing. Anal Chem; 82: 4158–64 20415489 10.1021/ac100341n

[CIT0023] Kong F (2009) Development of cellulosic paper-based test stripts for Cr (VI) determination. BioResources; 4: 1088

[CIT0024] Kong F Ni Y (2009) Determination of Cr(VI) concentration in diluted samples based on the paper test strip method. Water Sci Technol; 60: 3083–9 19955631 10.2166/wst.2009.741

[CIT0025] Kunimasa K Arita M Tachibana H (2011) Chemical pneumonitis and acute lung injury caused by inhalation of nickel fumes. Intern Med; 50: 2035–8 21921392 10.2169/internalmedicine.50.5557

[CIT0026] Lafleur JP Senkbeil S Jensen TG (2012) Gold nanoparticle-based optical microfluidic sensors for analysis of environmental pollutants. Lab Chip; 12: 4651–6 22824920 10.1039/c2lc40543a

[CIT0027] Leman A Omar A Yusof M (2010) Monitoring of welding work environment in small and medium industries (SMIs). Int J Res Rev Appl Sci; 5: 18–26

[CIT0028] Li M Tian J Al-Tamimi M (2012) Paper-based blood typing device that reports patient’s blood type “in writing”. Angew Chem Int Ed Engl; 51: 5497–501 22511466 10.1002/anie.201201822

[CIT0029] Liu XY Cheng CM Martinez AW (2011) A portable microfluidic paper-based device for ELISA. Institute of Electrical and Electronics Engineers 24th International Conference on Micro Electro Mechanical Systems (MEMS) 23–7 January Cancun, Mexico

[CIT0030] López Marzo AM Pons J Blake DA (2013) All-integrated and highly sensitive paper based device with sample treatment platform for Cd^2+^ immunodetection in drinking/tap waters. Anal Chem; 85: 3532–8 23445438 10.1021/ac3034536

[CIT0031] ‘t Mannetje A Brennan P Zaridze D (2012) Welding and lung cancer in Central and Eastern Europe and the United Kingdom. Am J Epidemiol; 175: 706–14 22343633 10.1093/aje/kwr358

[CIT0032] Martinez AW Phillips ST Carrilho E (2008) Simple telemedicine for developing regions: camera phones and paper-based microfluidic devices for real-time, off-site diagnosis. Anal Chem; 80: 3699–707 18407617 10.1021/ac800112r PMC3761971

[CIT0033] Martinez AW Phillips ST Whitesides GM (2010) Diagnostics for the developing world: microfluidic paper-based analytical devices. Anal Chem; 82: 3–10 20000334 10.1021/ac9013989

[CIT0034] Mehta S Shende R Sampat B (2012) A rare cause of respiratory distress. Chest; 142: 1002A

[CIT0035] Mentele MM Cunningham J Koehler K (2012) Microfluidic paper-based analytical device for particulate metals. Anal Chem; 84: 4474–80 22489881 10.1021/ac300309c

[CIT0036] Nelson DI Concha-Barrientos M Driscoll T (2005) The global burden of selected occupational diseases and injury risks: methodology and summary. Am J Ind Med; 48: 400–18 16299700 10.1002/ajim.20211

[CIT0037] Penner EM Inman WR (1963) Determination of copper in high-purity niobium, tantalum, molybdenum and tungsten metals with bathocuproïne. Talanta; 10: 407–12 10.1016/0039-9140(66)80067-x 18959902

[CIT0038] Phillips JI Green FY Davies JC (2010) Pulmonary and systemic toxicity following exposure to nickel nanoparticles. Am J Ind Med; 53: 763–7 20623660 10.1002/ajim.20855

[CIT0039] Quansah R Jaakkola JJ (2009) Paternal and maternal exposure to welding fumes and metal dusts or fumes and adverse pregnancy outcomes. Int Arch Occup Environ Health; 82: 529–37 18820944 10.1007/s00420-008-0349-6

[CIT0040] Rasband WS ImageJ, U. S. National Institutes of Health, Bethesda, Maryland, USA, http://imagej.nih.gov/ij/, 1997–2012

[CIT0041] Ratnarathorn N Chailapakul O Henry CS (2012) Simple silver nanoparticle colorimetric sensing for copper by paper-based devices. Talanta; 99: 552–57 22967593 10.1016/j.talanta.2012.06.033

[CIT0042] Rattanarat P Dungchai W Cate DM (2013) A microfluidic paper-based analytical device for rapid quantification of particulate chromium. Anal Chim Acta; 800: 50–5 24120167 10.1016/j.aca.2013.09.008 PMC3842604

[CIT0043] Sameenoi Y Panymeesamer P Supalakorn N (2013) Microfluidic paper-based analytical device for aerosol oxidative activity. Environ Sci Technol; 47: 932–40 23227907 10.1021/es304662w PMC3556395

[CIT0044] Su J Al-Tamimi M Garnier G (2012) Engineering paper as a substrate for blood typing bio-diagnostics. Cellulose; 19: 1749–58

[CIT0045] Szram J Schofield SJ Cosgrove MP (2013) Welding, longitudinal lung function decline and chronic respiratory symptoms: a systematic review of cohort studies. Eur Respir J; 42: 1186–93 23258779 10.1183/09031936.00206011

[CIT0046] US Department of Labor (USDOL) (2007) Welding, cutting, and brazing. Available at https://www.osha.gov/SLTC/weldingcuttingbrazing/. Accessed 02 August 2013.

[CIT0045a] US Department of Labor (USDOL) (2008) Metal and metalloid particulates in workplace atmospheres (ICP analysis). Available at https://www.osha.gov/dts/chemicalsampling/data/CH_276100.html Accessed 02 August 2013.

[CIT0048] Wang S Ge L Song X (2012) Simple and covalent fabrication of a paper device and its application in sensitive chemiluminescence immunoassay. Analyst; 137: 3821–7 22773999 10.1039/c2an35266d

[CIT0049] Wilkins DH Frederick Smith G (1953) 2,6-Bis(2-pyridyl)pyridine and alkyl derivatives. Their properties in the formation of ferrous and cobaltous colored complex cations. Anal Chim Acta; 9: 338–48

[CIT0050] Xiao J Meng YY Zhang PL (2012) Quantitative analysis of chromate (CrVI) by normal Raman spectroscopy and surface-enhanced Raman spectroscopy using poly(diallyldimethylammonium) chloride-capped gold nanoparticles. Laser Phys; 22: 1481–88

[CIT0051] Xu J Che P Ma Y (1996) More sensitive way to determine iron using an iron(II)-1,10-phenanthroline complex and capillary electrophoresis. J Chromatogr A; 749: 287–94 8921599 10.1016/0021-9673(96)00457-8

[CIT0052] Yang X Wang E (2011) A nanoparticle autocatalytic sensor for Ag^+^ and Cu^2+^ ions in aqueous solution with high sensitivity and selectivity and its application in test paper. Anal Chem; 83: 5005–11 21591668 10.1021/ac2008465

[CIT0053] Yang YK Yook KJ Tae J (2005) A rhodamine-based fluorescent and colorimetric chemodosimeter for the rapid detection of Hg^2+^ ions in aqueous media. J Am Chem Soc; 127: 16760–1 16316202 10.1021/ja054855t

[CIT0054] Zeidler-Erdely PC Battelli LA Salmen-Muniz R (2011) Lung tumor production and tissue metal distribution after exposure to manual metal ARC-stainless steel welding fume in A/J and C57BL/6J mice. J Toxicol Environ Health A; 74: 728–36 21480047 10.1080/15287394.2011.556063

[CIT0055] Zhao W Ali MM Aguirre SD (2008) Paper-based bioassays using gold nanoparticle colorimetric probes. Anal Chem; 80: 8431–7 18847216 10.1021/ac801008q

[CIT0056] Zwanenburg P Li X Liu XY (2013) Magnetic valves with programmable timing capability for fluid control in paper-based microfluidics. Institute of Electrical and Electronics Engineers 26th International Conference on Micro Electro Mechanical Systems (MEMS) 20–4 January, Taipei, Taiwan

