# Supplementary material for: Rapid Detection of Transition Metals in Welding Fumes Using Paper-Based Analytical Devices
Source: Ann Occup Hyg. 2014 Feb 10;58(4):413–23. doi: 10.1093/annhyg/met078 (PMC3979282; doi:10.1093/annhyg/met078)
Supplement: Supplementary Data [file supp_58_4_413__index.html]

Rapid Detection of Transition Metals in Welding Fumes Using Paper-Based Analytical Devices — Rapid Detection of Transition Metals in Welding Fumes Using Paper-Based Analytical Devices — Supplementary Data 

# Rapid Detection of Transition Metals in Welding Fumes Using Paper-Based Analytical Devices

## Supplementary Data

Data files

**Files in this Data Supplement:**

- Supplementary Data - Supplementary Data
