# Supplementary material for: Rapid Detection of Transition Metals in Welding Fumes Using Paper-Based Analytical Devices
Source: Ann Occup Hyg. 2014 Feb 10;58(4):413–23. doi: 10.1093/annhyg/met078 (PMC3979282; doi:10.1093/annhyg/met078)
Supplement: Supplementary Data [file supp_met078_Cate_Supplementary_Material.docx]

SUPPLEMENTARY MATERIAL FOR

**Rapid Detection of Transition Metals in Welding Fumes Using**

**Paper-Based Analytical Devices**

David M. Cate, Pavisara Nanthasurasak, Pornpak Riwkulkajorn , Christian L’Orange , Charles S. Henry*, John Volckens*

**
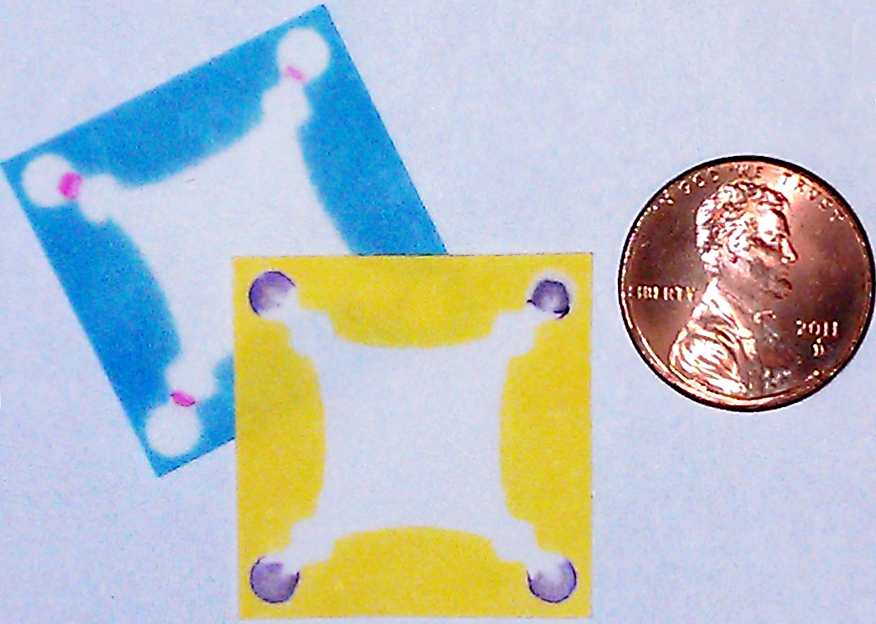
**

**Figure S1.** Comparing the size of two PAD devices to a penny.

**Figure S2.** Inter-device variability for Fe (top) and Ni (bottom) detection over three to four weeks. For Fe and Ni, the average difference in the slope for all linear regressed fits was 4.8 ± 4.4 % and 9.7 ± 5.2 % respectively. The average difference in measured intensity per mass of metal for Fe and Ni was 5.6 ± 5.7 and 1.5 ± 0.59 respectively.


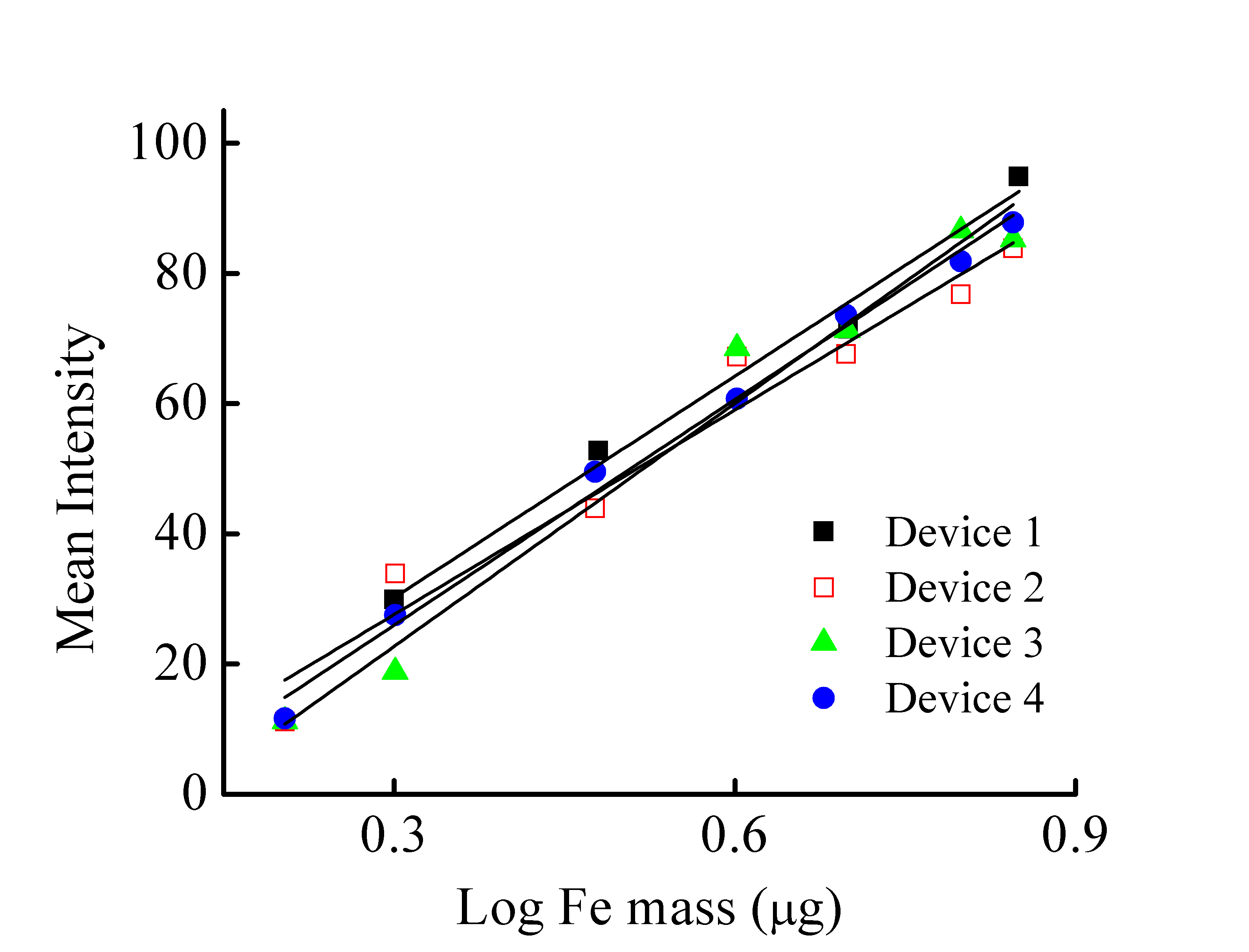

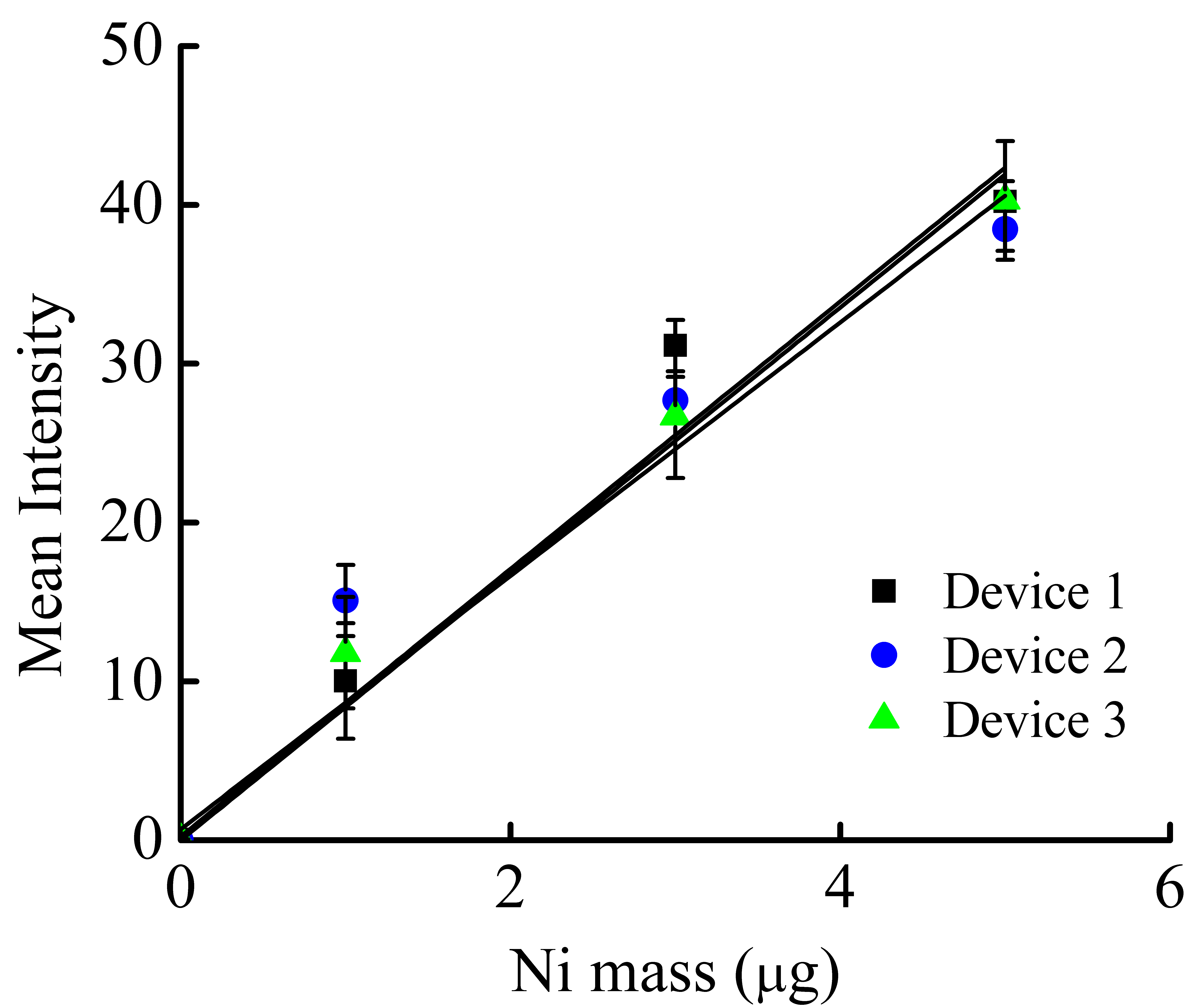


| Analyte | µPAD Signal Intensity Range (PIU^a^ ± SD) | Color Hue Thresholding Window Applied | TWA^b^ Detection Range (µg m^-3^ ± SD) |
| --- | --- | --- | --- |
| Fe | 18.7-121 ± 6.5 | 18 - 230 | 7.80-107 ± 6.9 |
| Cu | 27.0-79.3 ± 5.6 | 35 - 225 | 10.7-121 ± 8.9 |
| Ni | 19.1-48.0 ± 5.6 | 10 - 210 | 7.80-64.2 ± 5.8 |
| Cr | 17.7-87.9 ± 3.7 | 0 - 180 | 2.66-42.8 ± 3.9 |
| ^a^ Pixel Intensity Unit | |  |  |
| ^b^ Time Weighted Average | |  |  |

**Table S1.** Experimental conditions vs. signal intensity.
